# Classification of drug molecules for oxidative stress signalling pathway

**DOI:** 10.1049/iet-syb.2018.5078

**Published:** 2019-08-02

**Authors:** Nikhil Verma, Harpreet Singh, Divya Khanna, Prashant Singh Rana, Sanjay Kumar Bhadada

**Affiliations:** ^1^ Computer Science and Engineering Department Thapar Institute of Engineering and Technology Patiala Punjab 147004 India; ^2^ Department of Endocrinology Postgraduate Institute of Medical Education and Research Chandigarh 160012 India

**Keywords:** cancer, cellular biophysics, biochemistry, drugs, molecular biophysics, proteins, learning (artificial intelligence), medical computing, oxidative stress, Nrf2‐antioxidant response element signalling pathway, ARE signalling pathway, diabetes, cancer, hypertension, Alzheimers’ disease, heart failure, machine learning techniques, K‐fold cross‐validation method, ARE molecules

## Abstract

In humans, oxidative stress is involved in the development of diabetes, cancer, hypertension, Alzheimers’ disease, and heart failure. One of the mechanisms in the cellular defence against oxidative stress is the activation of the Nrf2‐antioxidant response element (ARE) signalling pathway. Computation of activity, efficacy, and potency score of ARE signalling pathway and to propose a multi‐level prediction scheme for the same is the main aim of the study as it contributes in a big amount to the improvement of oxidative stress in humans. Applying the process of knowledge discovery from data, required knowledge is gathered and then machine learning techniques are applied to propose a multi‐level scheme. The validation of the proposed scheme is done using the K‐fold cross‐validation method and an accuracy of 90% is achieved for prediction of activity score for ARE molecules which determine their power to refine oxidative stress.

## 1 Introduction

Stress is broadly defined as a noxious factor (physical, chemical or biological), which triggers a series of cellular and systemic events, resulting in the restoration of cellular and organismal homeostasis [1]. To cope with conditions of stress, organisms have developed stress response mechanisms, acting at the cellular or organelle‐specific level. The cellular stress response is a wide range of molecular changes that cells undergo in response to environmental stressors including extremes of temperature, exposure to toxins and mechanical damage [2, 3, 4].

Cellular stress responses are primarily mediated through what are classified as stress proteins. The cellular stress response pathway is based on the induction of cytoprotective proteins the so‐called stress proteins [5]. One such signalling pathway is Nrf2‐antioxidant response element (ARE) signalling pathway [6].

**Fig. 1:**
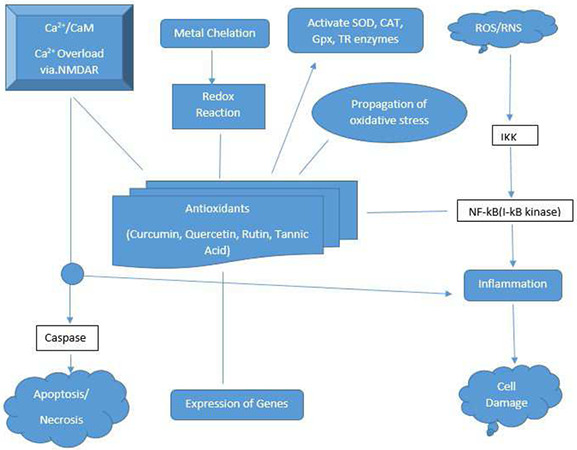
Antioxidant defence against free radical induced damage in a human body

The ARE possesses structural and biological features that characterise its unique responsiveness to oxidative stress. It is activated not only in response to H_2_O_2_ but specifically by chemical compounds with the capacity to either undergo redox cycling or be metabolically transformed to a reactive or electrophilic intermediate as shown in Fig. 1 [7].

The process of oxidation in the human body damages cell membranes and other structures including cellular proteins, lipids, and DNA. When oxygen is metabolised, it creates free radicals, which steal electrons from other molecules, causing damage [8].

**Fig. 2:**
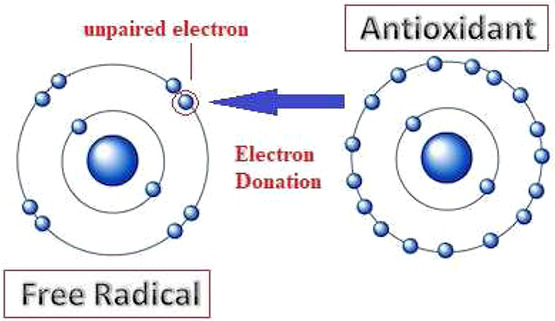
Neutralisation of free radical by antioxidant

Oxidative stress is essentially an imbalance between the production of free radicals and the ability of the body to counteract or detoxify their harmful effects through neutralisation by antioxidants as shown in Fig. 2.

A major mechanism in the cellular defence against oxidative or electrophilic stress is the activation of the Nrf2‐ARE signalling pathway, which controls the elimination of reactive oxidants by enhancing cellular antioxidant capacity [7].

**Fig. 3:**
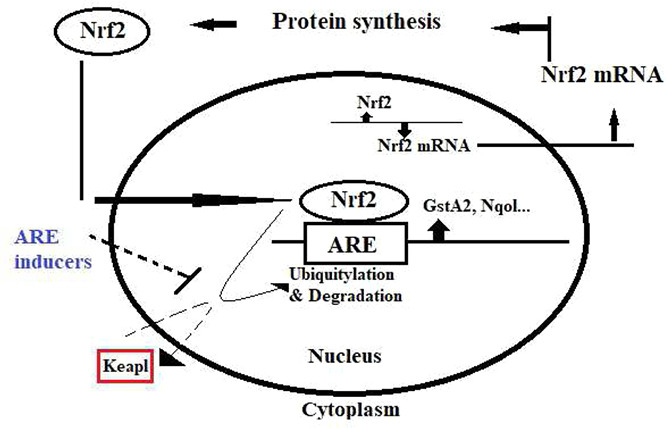
Nrf2‐antioxidant signalling pathway

Oxidative stress has been implicated in the pathogenesis of a variety of diseases ranging from cancer to neurodegeneration [9]. The ARE signalling pathway plays an important role in the amelioration of oxidative stress as shown in Fig. 3.

Machine learning (ML) provides methods, techniques, and tools that can help to solve diagnostic and prognostic problems in a variety of medical domains [10]. ML is being used for the analysis of the importance of clinical parameters and their combinations for prognosis, e.g. prediction of disease progression, extraction of medical knowledge for outcome research, therapy planning, and support and for the overall patient management [11].

ML methods can help the integration of computer‐based systems in the health care environment providing opportunities to facilitate and enhance the work of medical experts and ultimately to improve the efficiency and quality of medical care [12]. ML is already being used in the field of genomics [13]. Modern biology allows the high‐throughput measurement of many cell variables, including gene expression, splicing, and proteins binding to nucleic acids [10].

Over the past several decades, ML tools, such as quantitative structure activity relationship modelling, were developed that can identify potential biological active molecules from millions of candidate compounds quickly and cheaply [14]. Computational tools have been developed and applied to drug discovery as cost‐effective alternatives to traditional experiment protocols. The accurate identification of new hits from large chemical libraries by computational models is desirable for the pharmaceutical industry because it can reduce the costs and time associated with experiments needed to obtain new drug candidates with optimised pharmacodynamics and pharmacokinetic properties [15]. ML has been used to predict levels of oxidative stress in human subjects [16].

Computational strategies have been used to generate novel molecules with good affinity to the desired biological target [17]. Numerous ML techniques such as neural networks, support vector machines (SVMs), random forests etc., have been used in the past for detecting drugs useful in curing diseases [18]. Small‐molecule drug discovery has been viewed as a challenging multidimensional problem in which various characteristics of compounds including efficacy, pharmacokinetics, and safety need to be optimised in parallel to provide drug candidates and for such tasks, artificial intelligence tools have been proved to be handy [19].

Molecular classification using ML has been in trend for the past many years. Conotoxins are disulphide‐rich small peptides, which are invaluable peptides that target ion channel and neuronal receptors. Conotoxins have been demonstrated as potent pharmaceutical in the treatment of a similar kind of disease as targeted by ARE, such as Alzheimers’ disease, Parkinson's disease, and others. ML‐based computational tool for efficiently and effectively recognising conotoxin types based on sequence information has been used in [20]. Similarly, a novel method based on binomial distribution and radial basis function network to predict the types of ion‐channel targeted conotoxins have been presented here [21].

In another research, an evaluation platform was developed using novel and statistically robust ternary models via different ML models (i.e. linear discriminant analysis, classification and regression tree, and SVMs). The platform is aimed at effectively classifying chemicals with agonistic, antagonistic, or no oestrogen receptor activities [22].

Looking at the role of ML in the medical domain, it motivates us to use ML methods and algorithms that can be applied to calculate the activity of ARE signalling pathway as it has already been used to predict oxidative stress in chronic inflammatory diseases [16].

The aim of this study is to propose a multi‐level prediction scheme to calculate the activity score, potency score, and efficacy score of ARE stress signalling pathway, which contributes in a big amount to the improvement of oxidative stress in humans.

## 2 Materials and methods

### 2.1 Data set and its features

The Knowledge Discovery from Database generally abbreviated as KDD process is a data mining process of discovering interesting knowledge from a large amount of data stored in databases or other information repositories [23]. The KDD process consists of an iterative sequence of the following steps:

- Data integration: where multiple data sources may be combined
- Data cleaning: to remove noise and inconsistent data
- Data selection: where data relevant to the analysis task is retrieved from the database
- Data mining: an essential process where intelligent methods are applied in order to extract patterns
- Knowledge representation: to visualise the knowledge so obtained

The unbalanced dataset chosen in the study consists of active and inactive molecules of the ARE stress response pathway has been taken from [24]. These are available in the form of. *sdf* file extension format and data with features were extracted from the same using the tool PaDEL Descriptor [25]. PaDEL is a software to calculate molecular descriptors and fingerprints.

The data so decoded using the defined tool consists of 1444 1D and 2D features and decoded value is noted in a file. The brief description of a few features is given in Table 1.

**Table 1:** Molecular descriptors calculated by PaDEL

Descriptor type	Descriptor ID	Class
AcidicGroupCount	nAcid	2D
ALOGP	ALogP, ALogP2, AMR	2D
APol	Apol	2D
aromatic atoms count	naAromAtom	2D
aromatic bonds count	nAromBond	2D
atom count	nAtom, nHeavyAtom, nH, nB, nC, nN, nO, nS, nP, nF, nCl, nBr, nI	2D
BasicGroupCount	nBase	2D
BondCount	nBonds, nBonds2, nBondsS, nBondsS2, nBondsS3, nBondsD, nBondsD2, nBondsT, nBondsQ	2D
BPol	Bpol	2D
carbon types	C1SP1, C2SP1, C1SP2, C2SP2, C3SP2, C1SP3, C2SP3, C3SP3, C4SP3	2D
HBondAcceptorCount	nHBAcc, nHBAcc2, nHBAcc3, nHBAcc_Lipinski	2D
HBondDonorCount	nHBDon, nHBDon_Lipinski	2D
LargestChain	nAtomLC	2D
LargestPiSystem	nAtomP	2D
LongestAliphaticChain	nAtomLAC	2D
MannholdLogP	MLogP	2D
McGowanVolume	McGowan_Volume	2D
MLFER	MLFER_A, MLFER_BH, MLFER_BO, MLFER_S, MLFER_E, MLFER_L	2D
ring count	nRing, n3Ring, n4Ring, n5Ring, n6Ring, n7Ring, n8Ring, n9Ring, n10Ring, n11Ring, n12Ring, nG12Ring, nFRing, nF4Ring, nF5Ring, nF6Ring, nF7Ring, nF8Ring, nF9Ring, nF10Ring, nF11Ring, nF12Ring, nFG12Ring, nTRing, nT4Ring, nT5Ring, nT6Ring, nT7Ring, nT8Ring, nT9Ring, nT10Ring, nT11Ring, nT12Ring, nTG12Ring	2D
rotatable bonds count	nRotB	2D
rule of five	LipinskiFailures	2D
topological polar surface area	TopoPSA	2D
van der Waals volume	VABC	2D
weight	MW	2D
XLogP	XLogP	2D
charged partial surface area	PPSA‐1, PPSA‐2, PPSA‐3, PNSA‐1, PNSA‐2, PNSA‐3, DPSA‐1, DPSA‐2, DPSA‐3, FPSA‐1, FPSA‐2, FPSA‐3, FNSA‐1, FNSA‐2, FNSA‐3, WPSA‐1, WPSA‐2, WPSA‐3, WNSA‐1, WNSA‐2, WNSA‐3, RPCG, RNCG, RPCS, RNCS, THSA, TPSA, RHSA, RPSA	3D
moment of inertia	MOMI‐X, MOMI‐Y, MOMI‐Z, MOMI‐XY, MOMI‐XZ, MOMI‐YZ, MOMI‐R	3D
Pubchem fingerprint	Hierarchal element countsRings in a canonic extended smallest set of smallest rings ring setSimple atom pairsSimple atom nearest neighboursDetailed atom neighbourhoodsSimple SMARTS patternsComplex SMARTS patterns	fingerprint

Activity information of total 10,486 molecules was chosen, which include active, inactive and inconclusive molecules. Inactive molecules have an activity score as 0, inconclusive molecules do have an activity score of 30 and active molecules do have their activity score ranging from 40 to 100 (integral value). Both inconclusive and active molecules have their potency varying from 0 to 68.59 and efficacy score ranging from 15 to 490 were inconclusive molecules generally lie on the lower side of the band and opposite for the active molecules [26].

### 2.2 Data pre‐processing

Preprocessing the data chosen for study include some phases of the KDD process and some other important techniques described briefly below.

#### 2.2.1 Data integration

The chosen data include two databases one having the data related to active molecules while another having inactive molecules. Both the databases were then merged to get a single large database, which can be used for model building and training process in subsequent phases. After this phase, the dataset consists of 7149 tuples and 1444 attributes.

#### 2.2.2 Dimensionality reduction using FSelector

Dimensionality reduction or feature selection is the process of narrowing down a subset of features or attributes to be used in the predictive modelling process [27]. Feature selection is useful on a variety of fronts: it is the best weapon against the curse of dimensionality.

In this study, the FSelector [28] is available under License GPL‐2 and defines functions for selecting attributes from a given dataset. Attribute subset selection is the process of identifying and removing as much of the irrelevant and redundant information as possible. The formula representing target as an equivalent of selected attributes is shown as

$$\begin{array}{rl} \text{Activity} & =f(\text{naAromAtom},\text{ATS}0m,\text{AATS}7v,\ldots ,\\ & \;n3\text{HeteroRing},\text{VR}3\_D,\text{AMW}) \end{array}$$

The filter method for feature selection used here is cfs [29], in which the algorithm finds attribute subset using correlation and entropy measures for continuous and discrete data. The algorithm makes use of *best*–*first*–*search* for searching the attribute subset space. Doing dimensionality reduction the dataset now contains 7149 records having 27 attributes.

#### 2.2.3 Data cleaning by removing missing values

The data so obtained is not clean in the sense that it consists of missing values. The tuples having missing values are ignored for further consideration. This is usually done when a class label is missing or some attributes’ value is not defined. After this phase, the dataset consists of 6504 records and 27 attributes. In this dataset, 1084 records are active molecules and 5420 are inactive molecules.

#### 2.2.4 Balancing the dataset

Imbalanced class distribution is a scenario where the number of observations belonging to one class is significantly lower than those belonging to the other class. Same is the problem with the dataset in hand, as molecules which are active (minority class) are far less in number compared to inactive molecules (majority class). To handle this problem two techniques are applied:

- Oversampling: It was done by increasing the frequency of minority class (active molecules). The synthetic minority over‐sampling technique (SMOTE) algorithm is one of the first and still the most popular algorithmic approach to generating new dataset samples. The algorithm works by oversampling the underlying dataset with new synthetic points [30]. The SMOTE algorithm is parameterised with *k*‐neighbours and the number of new points you wish to create. Each step of the algorithm will
  (i) Randomly select a minority point.
  (ii) Randomly select any of its *k*‐neighbours belonging to the same class.
  (iii) Randomly specify a lambda value (constant required during the procedure) in the range [0, 1].
  (iv) Generate and place a new point on the vector between the two points, located lambda per cent of the way from the original point.
- The detailed algorithmic steps are mentioned in [30]. Chose *k*‐neighbours as 50 and number of new points to be created as 4336.
- Applying the oversampling technique, only 4336 new entities have been created during oversampling so that minority and majority classes come equally in number. The dataset is divided into five balanced sub‐datasets as entities belonging to the majority class are five times in number as compared to minority class entities.
- Each sub‐dataset having an equal number of active and inactive molecules to train the prediction model and then ensemble the results to get an overall final prediction.
- Undersampling: It was done by decreasing the frequency of the majority class (inactive molecules.) Applying the undersampling technique, the dataset is chosen by randomly selecting majority class samples equal in number as that of minority class samples, so as to get a balanced dataset.

### 2.3 ML methods

In this work, six ML models are used for prediction of the activity of various molecules. All five sub‐datasets after balancing are trained using these models and an overall consensus is derived using the ensembling technique with equal weightage given to results obtained from every sub dataset [31]. The models are available in R open source software. R is licensed under GNU GPL. These models used with available package in R statistical tool along with their tuning parameters are shown in Table 2.

**Table 2:** ML models used for classification of molecules

	Model	Method	Package	Tuning Parameter(s)	Ref.
M1	ada boost	ada	kernlab, rpart, ada, hmeasure	maxdepth, cp, minsplit, xval, iter	[32]
M2	decision tree	rpart	rpart, hmeasure	parms, control	[33]
M3	linear model	multinom	car, nnet, hmeasure	maxit	[34]
M4	neural network	nnet	nnet, hmeasure	size, MaxNWTs, maxit	[35]
M5	random forest	randomForest	randomForest, hmeasure	ntree, mtry	[36]
M6	SVM	ksvm	e1071, kernlab, heasure	rules, pruned, kernel	[37]

## 3 Methodology

The methodology followed by the proposed model is as follows:

- Data acquisition from [24].
- Feature extraction using PADEL descriptor [25].
- Data preprocessing using KDD process.

LEVEL 1:

- Training ML models for binary classification of the dataset into two classes (active or inactive).
- Testing trained models in the above step and evaluate using evaluation parameters for classification and in last, to find out the best model.

LEVEL 2:

- Training ML models for regression dataset of activity, potency and efficacy scores.
- Testing the proposed scheme.
- Result analysis.

**Fig. 4:**
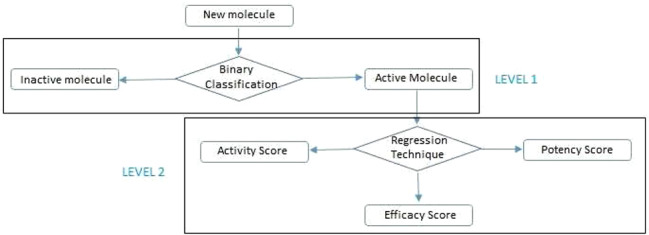
Multi‐level proposed prediction scheme for new molecules

For any new molecule, the work flow to find its class (active or inactive) using classification and then its activity, potency and efficacy score using the regression model would be as shown in Fig. 4.

## 4 Model evaluation parameters

Ideally, the estimated performance of a model tells how well it performs on unseen data, i.e. making predictions on future data. Various performance measures are

(i) Receiver operating characteristics (ROC)
(ii) *H*‐measure (*H*)
(iii) Gini coefficient (*G*)
(iv) Accuracy
(v) Correlation
(vi) Coefficient of determination (*R* ^2^)

### 4.1 Receiver operating characteristic (ROC)

The ROC curve, which is defined as a plot of test sensitivity (=TPR) as the *y*‐coordinate versus its 1‐specificity (=FPR) as the *x* coordinate is an effective method of evaluating the performance of diagnostic tests. Sensitivity and specificity, which are defined as

(1) sensitivity=TP/(TP+FN)

(2) specificity=TN/(FP+TN)

where TP is the number of true positive decisions, TN is the number of true negative decisions, FN is the number of false negative decisions and FP is the number of false positive decisions.

Several summary indices are associated with the ROC curve. One of the most popular measures is the area under the curve (AUC). AUC is a combined measure of sensitivity and specificity.

In binary classification, the class prediction for each instance is often made based on a continuous random variable *X*, which is a ‘score’ computed for the instance. Given a threshold parameter *T*, the instance is classified as ‘positive’ if X>T and ‘negative’ otherwise. *X* follows a probability density $f_{1}(x)$ if the instance actually belongs to class ‘positive’, and $f_{0}(x)$ if otherwise. Therefore, the true positive rate is given by:

(3) $$\text{TPR}(T)=\int_{T}^{\infty }f_{1}(x)dx$$

and the false positive rate is given by:

(4) $$\text{FPR}(T)=\int_{T}^{\infty }f_{0}(x)dx$$

The ROC curve plots parametrically TPR(*T*) versus FPR(*T*) with *T* as the varying parameter.

The AUC is given by (the integral boundaries are reversed as large *T* has a lower value on the *x*‐axis):

(5) $$A=\int_{\infty }^{-\infty }\text{TPR}(T)d(\text{FPR}(T))$$

It is used as an evaluation parameter for classification models.

### 4.2 *H*‐measure

The *H*‐measure is a measure of classification performance proposed in [38].

The threshold parameter *T* (as mentioned in Section 4.1) allows the end user to ‘tune’ a classier in order to trade‐off FPs for FNs or vice versa. An extreme example is where one classifies all objects as positive, for T=−∞ regardless of their description, enabling one to never ‘miss a case’ (FN = 0), at the cost of a large number of ‘false alarms’(high FP). Conversely, for T=∞, no objects will ever be classified as positive, forcing FP = 0 at the cost of incurring a maximum number of FNs.

The AUC has come under criticism for handling the aforementioned trade‐off in a fundamentally incoherent manner, in the sense that it treats the relative severities of misclassifications differently when different classifiers are used. A coherent alternative proposed known as the *H*‐measure that can optionally accommodate expert knowledge regarding misclassification costs, whenever that is available [38].

Most of the metrics discussed in Section 4.1 attempt to take a balanced view of the trade‐off between FPs and FNs. A principled way to achieve this is to introduce the notion of misclassification costs, which seek to quantify the relative severity of one type of error over the other. Let *c* in [0,1] denote the ‘cost’ of misclassifying a class 0 object as class 1 (i.e. FP), and 1−c the cost of misclassifying a class 1 object as class 0 (i.e. FN). Let the total cost be denoted by

(6) L(c;T)

It is realistic to specify a distribution instead, *w*(*c*), over different values of *c*, capturing the end user's uncertainty about the exact values of the costs

(7) $$L_{w}=\int_{c}L(c;T)w(c)dc$$

This notion of averaged minimum cost‐weighted loss allows formulating a criticism of the AUC which in turn motivates the *H*‐measure.

The *H*‐measure can be calculated from here as

(8) $$H=1-\frac{L_{w}}{L_{w}^{\max}}$$

where $L_{w}^{\max}$ represents the max value of $L_{w}$.

### 4.3 Gini coefficient

Gini coefficient (*G*) is a measure of statistical dispersion and inequality in the distribution is measured through the Gini coefficient. It is closely related to the AUC as

(9) AUC=(G+1)/2

It is used as an evaluation parameter for classification models.

### 4.4 Accuracy

The accuracy is calculated as the percentage deviation of predicted value (*p*) with the actual value (*a*) for *n* number of observations

(10) $$\begin{array}{rl} & \text{Accuracy}=\frac{100}{n}\sum_{i=1}^{n}q_{i}\\ & q_{i}=\left(\begin{array}{rl} 1 & \;\text{if}\;\;p_{i}=a_{i}\\ 0 & \;\text{otherwise} \end{array}\right. \end{array}$$

It is used as an evaluation parameter for classification models. While the formula for finding the accuracy of the regression model is

(11) $$\begin{array}{rl} & \text{Accuracy}=\frac{100}{n}\sum_{i=1}^{n}q_{i}\\ & q_{i}=\left(\begin{array}{rl} 1 & \;\text{if}\;\text{abs}(p_{i}-a_{i})\leq \text{error}\\ 0 & \;\text{otherwise} \end{array}\right. \end{array}$$

### 4.5 Correlation (*r*)

The relationship between two sets of variables used to describe or predict information is known as correlation. It is the degree to which the change in a set of variables is related. It is calculated as

(12) $$r=\frac{n\sum xy-\sum x\sum y}{\sqrt{\left[n\sum x^{2}-{\left(\sum x\right)}^{2}\right]\left[n\sum y^{2}-{\left(\sum y\right)}^{2}\right]}}$$

where *n* is the number of observations, *x* is the actual value and *y* is the predicted value. It is used as an evaluation parameter for regression models.

### 4.6 Coefficient of determination (*R* ^2^)

The coefficient of determination (*R* ^2^) summarises the explanatory power of the regression model for target value or data point *y* by the predictor *x* is computed from the sums‐of‐squares terms

(13) $$R^{2}=\frac{\text{SSR}}{\text{SST}}=1-\frac{\text{SSE}}{\text{SST}}$$

where

- SSR is the ‘regression sum of squares’ and quantifies how far the estimated regression line, $y^{\prime }$, is from the sample mean or $\bar{y}$

$$\text{SSR}=\sum (y^{\prime }-\bar{y}){\;}^{2}$$

- SSE is the ‘error sum of squares’ and quantifies how much the data points, *y*, vary around the estimated regression line, $y^{\prime }$

$$\text{SSE}=\sum (y-y^{\prime }){\;}^{2}$$

- SST is the ‘total sum of squares’ and quantifies how much the data points, *y*, vary around their mean, $\bar{y}$

$$\text{SST}=\sum (y-\bar{y}){\;}^{2}$$

*R* ^2^ describes the proportion of variance of the dependent variable explained by the regression model. If the regression model is perfect, SSE is zero, and *R* ^2^ is 1. If the regression model is a total failure, SSE is equal to SST, no variance is explained by the regression, and *R* ^2^ is zero. It is used as an evaluation parameter for regression models.

## 5 K‐fold cross validation

A large number of comparisons are always preferred to compare the performance of the model. To run K‐fold cross validation multiple times or increase the number of comparisons, repeated K‐fold cross validation is useful. In K‐fold cross‐validation, only *k* comparisons are performed. In cross‐validation, in each fold, random data is provided to do the comparisons. Here, ten‐fold cross‐validation is repeated for three times.

## 6 Results

The ML models used for binary classification as described in Table 2 are trained on the dataset obtained from [24] and evaluated for the parameters discussed in Section 4. These parameters of various models calculated for the dataset used in the study are shown in Table 3.

**Table 3:** Evaluation results of models used for binary classification by oversampling

	Model	AUC	H‐measure	Gini	Accuracy, %
M1	ada boost	0.849	0.4328	0.6976	62.5
M2	decision tree	0.7502	0.26328	0.5002	53.0
M3	linear model	0.7634	0.3202	0.5268	51.5
M4	neural network	0.7162	0.21	0.4322	48.5
M5	random forest	0.8608	0.47	0.7216	72.0
M6	SVM	0.8248	0.3792	0.649	55.0

It can be easily analysed from Tables 3 and 4 that the random forest model outperforms all other models for the dataset chosen in the study, with the highest AUC (0.8608 and 0.862), *H*‐measure (0.47 and 0.451), Gini coefficient (0.7216 and 0.723) and accuracy (72% and 79.2%) (respectively, in oversampling and under‐sampling results). In terms of accuracy, the random forest model is then followed by Ada boost (62.5% and 75.0%) and SVM (55% and 72.29%).

**Table 4:** Evaluation results of models used for binary classification by undersampling

	Model	AUC	H‐measure	Gini	Accuracy, %
M1	ada boost	0.836	0.385	0.672	75.0
M2	decision tree	0.743	0.233	0.486	70.86
M3	linear model	0.777	0.286	0.554	71.01
M4	neural network	0.739	0.222	0.478	68.1
M5	random forest	0.862	0.451	0.723	79.2
M6	SVM	0.796	0.32	0.591	72.39

**Fig. 5:**
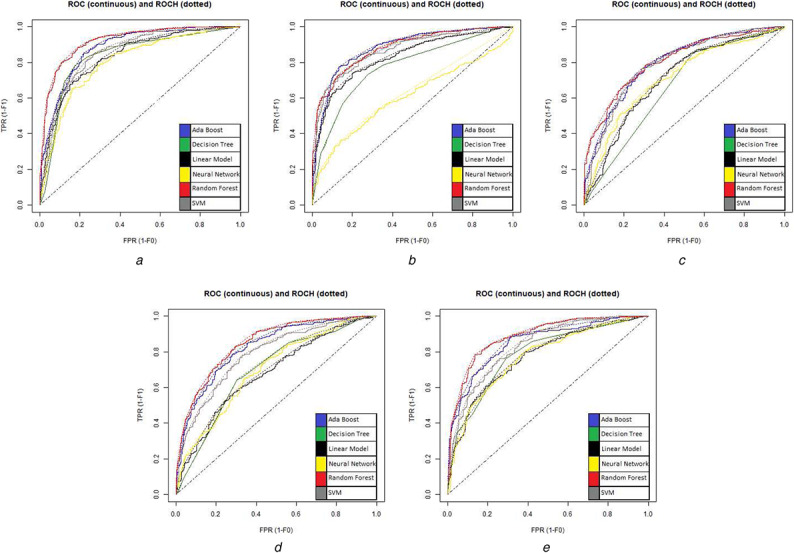
ROC curves of models used for binary classification by oversampling ***(a)*** ROC curve first sub‐dataset, ***(b)*** ROC curve second sub‐dataset, ***(c)*** ROC curve third sub‐dataset, ***(d)*** ROC curve fourth sub‐dataset, ***(e)*** ROC curve fifth sub‐dataset

**Fig. 6:**
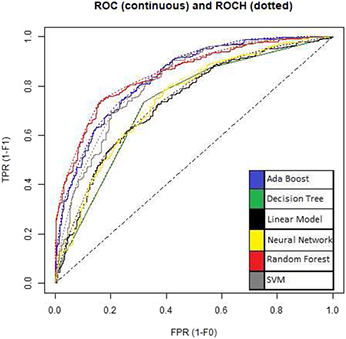
ROC curves of models used for binary classification by under‐sampling

The ROC curves for all the models used in the study are shown in Figs. 5 and 6. A test with perfect discrimination (no overlap in the two distributions) has a ROC curve that passes through the upper left corner (100% sensitivity, 100% specificity). Therefore the closer the ROC curve is to the upper left corner, the higher the overall accuracy of the test.

The accuracy of other classifiers is low compared to the random forest classifier. However, measures such as sensitivity and specificity are also important criteria in developing models for imbalanced datasets. Hence, the ROC curves of other classifiers need to be compared to the ROC curve for the random forest. ROC curves for all the models used are shown in Figs. 5 and 6 using a colouring scheme mentioned in graphs. It can be analysed from the curves that the random forest's curve is most upper left compared to curves of other models in nearly all the graphs.

Attributing to the same characteristic of the graphs, it can be analysed from Fig. 5 using Table 5 that ROC curve of the random forest with maximum AUC is obtained for Fig. 5*a* followed by Figs. 5*b*, e, d and *c*, which implies the order of how well trained the obtained models are on the dataset considered for oversampling.

**Table 5:** AUC values for red curves in Fig. 5

Curve	Fig. 5*a*	Fig. 5*b*	Fig. 5*c*	Fig. 5*d*	Fig. 5*e*
AUC	0.905	0.895	0.808	0.830	0.866

Chosen dataset enlist both active and inactive molecules. Using classification technique of ML, it is concluded that for a dataset of ARE molecules random forest model is the best model with the highest accuracy to predict the class of activity for any new molecule to be tested.

Table 6 describes the average root‐mean‐square error (RMSE), *R* ^2^ and mean absolute error (MAE) of the proposed model. The RMSE has been recorded by applying ten‐fold cross‐validation three times.

**Table 6:** Cross‐validation results

mtry	RMSE	*R* ^2^	MAE
2	0.1655441	0.9145148	0.09410988
3	0.1640467	0.9148280	0.08907119
4	0.1642636	0.9139220	0.08748790

Every active molecule has some activity score associated with it. By training the random forest model for regression dataset of activity, potency and efficacy scores of active molecules one could predict the same parameters for any new molecule to be evaluated subject to its activeness using the multi‐level proposed scheme.

Three random forest models were trained on the dataset of activity information of 10,486 molecules described in Section 2.1 and found to have accuracy as mentioned in Table 7 with some other evaluation parameters. It can be observed that accuracy as high as 90% is achieved to predict the activity score for training data while for potency and efficacy the measure is 82.5 and 80%, respectively. Also, the highest correlation and *R* ^2^ are gained while predicting activity score (0.86) in comparison with potency (0.6) and efficacy (0.68).

**Table 7:** Regression model evaluation results for predicting activity, efficacy and potency score

	Model for	Correlation	*R* ^2^	Accuracy, %
1	activity score	0.86	0.74	90
2	potency score	0.6	0.36	82.5
3	efficacy score	0.68	0.46	80

Comparing the result obtained from the proposed multi‐level prediction scheme to the work done in past, it can be seen that an overall highest accuracy is obtained for SVM, in [22], was 76.6%, while a similar score obtained in [21] was 85.7%, whereas the overall score obtained in the work proposed in this study is 90%, along with the cross‐validation test, indicating that the method is superior to other state‐of‐the‐art methods.

## 7 Conclusion

Several drugs that stimulate the Nrf2‐ARE pathway are being studied for the treatment of diseases that are caused by oxidative stress. The proposed multi‐level prediction scheme best suits the aim to detect the activity of any new molecule of ARE signalling pathway which is validated by suitable evaluation parameters. If found active then a selected model is able to successfully predict the activity score of the molecule under consideration with an accuracy of 90%.

Interestingly, oxidative stress pathways are commonly found in advanced‐stage kidney tumours and are important factors to consider and potentially target when developing therapeutic approaches. The proposed scheme would be highly beneficial in detecting the drug's potential to tap the disease from spreading further and cure the same.
